# Effectiveness of Visual-Based Interventions on Understanding Cancer Information: A Systematic Review

**DOI:** 10.1177/10732748261446035

**Published:** 2026-04-27

**Authors:** Linda Siachalinga, Hansoo Kim, Karin Brochstedt Dieperink, Elisabeth Coyne

**Affiliations:** 1 School of Medicine and Dentistry, 63619 Griffith University, Gold Coast, Australia; 2 School of Nursing and Midwifery, 110927 Griffith University, Gold Coast, Australia; 3 Family Focused Healthcare Research Center (FaCe), Department of Clinical Research, University of Southern Denmark, Odense, Denmark; 4 Department of Oncology, Odense University Hospital, Odense, Denmark

**Keywords:** visual, intervention, health literacy, knowledge, comprehension, understanding, cancer

## Abstract

**Introduction:**

The aim of this study was to assess the effectiveness of visual-based education intervention on understanding cancer information.

**Methods:**

We conducted a systematic review across PubMed, Embase and Scopus electronic databases using Preferred Reporting Items for Systematic Reviews and Meta-analysis, in July 2025. Randomized and non-randomized studies measuring impact of visual-based education interventions on health literacy, knowledge, comprehension, or understanding in cancer were considered for inclusion. Quality assessment was conducted using the Mixed Methods Appraisal Tool (MMAT). A descriptive analysis of included studies was performed, and a meta-analysis was performed for Randomised clinical trials (RCTs).

**Results:**

Twenty-one studies were included in the review, 11 RCTs, seven non-RCTs, and three mixed methods design. The studies were conducted in seven countries across five continents. A high proportion of the included studies satisfied the MMAT quality assessment criteria. Visual-based interventions improved knowledge and comprehension compared to non-visual communication such as audio or basic text. In one study, video intervention increased knowledge with mean difference 3.96 ± 1.69 compared to pamphlet (1.76 ± 1.42). Crowd-figure-pictograms with numeric data increased knowledge by 2.42 compared to non-numeric controls (0.20). Furthermore, 3D printed models improved understanding compared to imaging alone (4.60-4.78/5 vs. 4.06-4.49/5), and visual-based treatment timelines improved comprehension compared to audio (0.84 vs 0.68). However, certain visual-based interventions had no impact over well-designed static materials in some contexts. Health literacy was a significant moderator of outcomes, and low health literacy was consistently associated with poor baseline knowledge and comprehension, regardless of visualization format.

**Conclusion:**

Visual-based education interventions may improve knowledge, comprehension and understanding, overcoming barriers imposed by limited health literacy, hence empowering patients to self-manage their health across various cancer contexts.

## Introduction

Understanding and addressing modifiable risk factors of cancer through prevention, early detection and effective management can reduce its impact.^1^ Decision making for personal health necessitates more than just the provision of information by healthcare professionals. It also requires tailored information to the individual patient’s level of comprehension, ensuring that communication is accessible and actionable.^2^ In high burden diseases like cancer, patients with limited health literacy have more difficulty in navigating the health care system and making informed decisions^3,4^ when compared to diseases with burden. Understanding health information during cancer enables individuals with lower health literacy to play an active role in improving their own health.^2,5^ As most cancer services are delivered through outpatient clinics,^6^ supporting the person affected with cancer requires innovative ways to enable them access and understand information.^7,8^

Health literacy is defined as the degree to which individuals can obtain, process, understand, and communicate health-related information needed to make informed health decisions.^5,9^ It is a multifaceted concept that has evolved from a narrow focus on basic reading and numeracy to focus on individual skills that encompass a dynamic interaction between an individual and the demands of health systems and society.^10-12^ It concerns the knowledge and competences of individuals to meet the complexes of health, including understanding the factors that influence health and knowledge of how to address these factors.^11^ Health literacy is linked to literacy and extends beyond basic reading and numeracy to include a broad set of skills encompassing knowledge, comprehension, self-care, communication skills, accessing, appraising and applying health information.^11,13^

Studies have shown that low health literacy is linked to poor health outcomes including lower implementation of preventative health and lower treatment adherence.^4,14^ Individuals with limited health literacy often struggle with navigating the health care environment, including accessing care across the cancer continuum resulting into errors, poor quality of care, and risks to patient safety.^15,16^ Attributing factors include reliance on written materials to communicate health information, such as doctors’ instructions, preventative guidance for healthy lifestyle choices, and medication dosage directions.^4,17^ With an estimated 750 million adults globally living with low literacy^18^ both low literacy and limited health literacy present significant barriers for health professionals in delivering effective care.^17^

Improving the understanding of health information through various educational interventions have been proven an effective way to increase a person’s ability to self-manage their health.^19-21^ A recent review found that visually enhanced education materials improve comprehension compared to traditional methods of oral or written material.^15,22^ Visual-based interventions are defined by the Centers for Disease Control and Prevention as images, videos, and similar tools used to communicate information about a specific topic and to simplify the comprehension process.^15,23^ Visualising health information through graphics, images, and videos enhances the communication of complex concepts, supports consumer understanding and contributes to improved patient safety and outcomes.^24^ The effectiveness of visual-based interventions may be due to increased interactivity and reduced reading efforts.^15,25^

While systematic reviews^3,15,16^ have assessed the effect of various interventions on health literacy, none of the reviews specifically assessed the effects of visual-based education interventions on health literacy in cancer. A review by Housten et al.^16^ revealed that education, communication, technology and multimodal strategies are commonly employed in cancer improving screening and comprehension. Galmari et al (2024) assessed the effectiveness of various visual-based interventions on health literacy in healthcare included eight cancer-based studies but did not comprehensively analyse for the impact of visual interventions in cancer.^15^

A recent umbrella review by Jetaini et al (2025) that included 10 systematic reviews of cancer education confirmed that health literacy interventions targeting patient education and communication are the most common.^3^ Similar to findings by Housten et al (2021), multimodal interventions showed positive effect in improving health literacy.^3^ Among the included reviews, none primarily assessed the effect of visual-based education interventions on health literacy in cancer patients.^3^

Building on these reviews, the present review aims to assess the effectiveness of visual-based education intervention on improving understanding of cancer.

## Methods

The systematic review was conducted in accordance with the Preferred Reporting Items for Systematic reviews and Meta-Analysis (PRISMA) statement (Supplementary File 1).^26^

### Search Strategy

An electronic search via title and abstract was conducted in PubMed, Embase and Scopus from database inception to 27^th^ July 2025. The search terms included ‘visual intervention OR visual education’ AND ‘health literacy OR understanding’ AND ‘cancer OR oncology’, with additional relevant keywords expanded using the Boolean Operator OR. The search strategy is attached as Supplementary File 2.

### Eligibility Criteria

Inclusion criteria: Studies that focused on visual-based education interventions in the context of cancer, see Table 1. Eligible studies were those that primarily measured health literacy or proximal outcomes to health literacy including comprehension, understanding and knowledge in the context of cancer prevention, screening, diagnosis and treatment as an outcome, and were conducted among adult patient populations. Randomised, non-randomised and mixed methods studies with an intervention component were considered.

**Table 1.** Eligibility Criteria

​	Inclusion criteria	Exclusion criteria
Study design	Randomized controlled studies	Non-published studies
	Non-randomized (quasi experiments)	Qualitative studies
	Mixed methods with an intervention component	Descriptive quantitative studies
	Published studies	Reviews, case studies, abstracts, theses and study protocols
		Studies for which the full article was not accessible
Population	Cancer in adults	Non-cancer context
Intervention	Visual-based education	Non-visual-based interventions
Outcomes	Health literacy	No outcome of interest
	Knowledge, comprehension, understanding	

Exclusion criteria: Studies that were not focused on cancer as the study area, as well as those that did not include any intervention or the outcome of interest. Additionally, studies with inappropriate designs such as abstracts, reviews, qualitative studies, cross-sectional studies, letters to the editor, commentaries, reports, and unpublished articles were excluded. Non-English studies and those without accessible full texts were also excluded from the review.

### Outcomes of Interest

In this review, the outcomes of focus included health literacy measured as health literacy, knowledge, comprehension, or understanding. Measuring health literacy directly may be challenging due to its multidimensional nature leading to reliance on proximal outcomes such as knowledge, comprehension and understanding often measured through study-specific validated questionnaires.^3,15^ Knowledge, comprehension, and understanding are fundamental measures of health literacy^11,16,27^ justified by the definition of health literacy as the capacity to obtain, process, and understand basic health information^11,28^ and their status as confirmed measurable outcomes in intervention research.^3,15,16^ Considering the concerns from previous studies to the lack of standardised measures and tools in reporting the outcomes,^3,15,16^ the findings and tools of measurement are presented as reported in the included studies.

### Selection Process

The results of the electronic search were imported to endnote^29^ where duplicates were removed. The studies were then transferred to Covidence^30^ for screening. After removal of duplicates identified by Covidence, screening of titles and abstract was done by one author, L.S. A hand search of the reference lists of included studies was also conducted to identify additional eligible studies. The full texts evaluation was conducted by two authors, EC and LS, and conflicts were resolved through discussion.

### Data Extraction and Analysis

The data extracted from included studies were author’s name, year of publication, country, study setting/population, study design, aim of study, intervention employed, and outcomes reported. Data extraction was done using a standard data extraction form in excel. Descriptive analysis was used to summarize study findings. Additionally, meta-analysis of randomised clinical trials (RCTs) was conducted in Review Manager 5.4.^31^

### Quality Assessment

Quality assessment of studies was conducted by two researchers (LS and EC) by referring to the Mixed Method Appraisal Tool (MMAT).^32^ Each study was assessed using the MMAT criteria based on its methodological design. For each criterion, a score of 1 was applied if yes, 0 if no or unclear, and 1 if the criteria did not apply to the study. The score for each study was determined by calculating the number of yes responses out of the total number of applicable criteria. While we calculated the average score for all studies, we endeavoured to provide a more detailed presentation of the ratings of each criterion to inform the quality of the included studies better as recommended.^32^

## Results

### Characteristics of Included Studies

**Figure 1.**
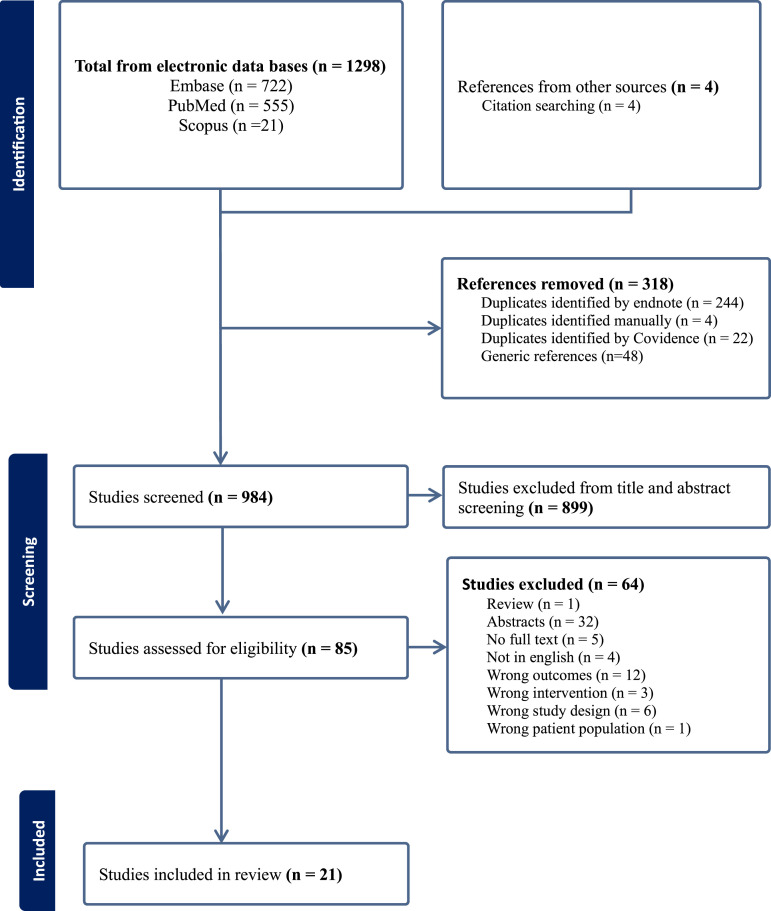
Prisma flow diagram

The systematic review included published studies of visual-based interventions in cancer. A total of 1298 studies were retrieved from search of electronic databases and four from reference searches. After removal of duplicates and generic references, 954 studies were considered for title and abstract screening, of which 85 studies were eligible for full-text screening. Finally, 21 studies were included in the final analysis (Figure 1).

The studies included were 11 RCTs, seven non-RCTs and three mixed methods in design. The studies were conducted across several countries, including the United States (12 studies), Australia and the Netherlands (two studies each), and one study each from the United Kingdom, China, Germany, Tanzania, and India. Population included patients with; prostate cancer (seven studies), three studies each for general cancer, cervical cancer, colorectal and breast cancer; and one study each for hematologic and skin cancer, see Table 2. The total population across all studies was 5,113, male, 2,054 (40.2%), female, 2,793 (54.6%), and unspecified, 266 (5.2%). The mean age was between 32.4 -83 years across all studies.

**Table 2.** Summary of the 21 Included Studies in a Systematic Review

Authors, year	Aim	Design	Sample	Cancer diagnosis	Outcomes and measuring tool	MMAT scores
Cooper, E. C 2021^33^	To report on the impact of intervening on baseline human papillomavirus and cervical cancer knowledge	Pre post surveys	764 women over 18 years	Cervical	Demographics, knowledge	100
Tanzania.			Mean age 36.4 (±11.1)		6 knowledge multiple choice question	*******
Frosch, D. L 2008^34^	To assess the effects of informational brochures and video decision aids about cancer screening on patient intention to engage in shared decision-making and its predictors in a racially diverse sample.	Quasi-experimental	207 men 50 years or older (45 or older for African American)	Prostate	Demographics, knowledge	100
USA			Mean age 61.3		Other outcomes: attitude, perceived social norms, self-efficacy, role preferences	*******
					Questionnaire	
Gao, J 2022^35^	To investigate the impact of a Virtual reality radiotherapy (RT) educational system on patients’ understanding and anxiety prior to commencing RT	Randomised controlled study	60 patients aged 18 to 75 years of receiving radiotherapy	General	Comprehension	100
China			36 men and 24 women		Other outcomes: anxiety	*******
			Mean age 50.63 ± 11.98		Questionnaire with 14 statements	
Gattellari, M 2005^36^	To compare the impact of three different information resources (leaflet, video, evidence-based booklet) about prostate cancer screening; to determine the extent to which men’s preferences for involvement in medical decision-making modified their impact, and to examine educational attainment as a possible effect modifier	Randomised controlled study	421 men between 50 and 70 years	Prostate	Demographics, knowledge	86
Australia			Mean age 57.8		Other outcomes: decisional conflict, decision control, worry, perceived ability to make informed choice, propensity to undergo screening, acceptability of doctor’s recommendation	******
					14-item knowledge measure	
Housten, A. J 2020^37^	Tested the effect of presentation format on comprehension of colorectal cancer screening probabilities to identify optimal risk communication strategies	Randomised controlled study	187 men and women aged 45 to 75 years	Colorectal	Demographics, knowledge	100
USA			118 women and 69 men		Other outcomes: perceived credibility	*******
			Median age 58 (IQR 14)		Questionnaire with 14 questions in total	
Ilic, D 2008^38^	To explore the effectiveness of presenting health information on prostate cancer screening using video, internet, and written interventions on patient decision making, attitudes, knowledge, and screening interest	Randomised controlled study	161 men aged over 45	Prostate	Demographics, knowledge	100
Australia			Mean age 56.7		Other outcomes: anxiety, decisional conflict, decision-making role, screening interest	*******
					A 5-item multiple-choice question	
Jambor, H. K 2025^39^	To develop and evaluate visual timelines for communicating cancer treatment paths using 3 haematological neoplasms as case studies	Mixed methods	496 participants	Haematological	Demographics, comprehension	100
Germany			91 men, 239 women and 166 unspecified		10 question questionnaires	*******
			Mean age 39.5			
Lewis, C. L 2010^40^	To develop and test a decision aid designed to promote individualized colorectal cancer screening decision making for adults age 75 and over.	Pre-post design	46 participants 75 years and older	Colorectal	Demographics, knowledge	86
USA			39 women and 7 men		A 15-item true/false questionnaire	******
			Mean age 83 (75-95)			
Menon, U 2008^41^	To describe the development and feasibility testing of a computer-based, theory-guided educational program designed to increase colorectal cancer screening	Mixed methods	199 participants aged above 50 years	Colorectal	Demographics, knowledge	100
USA			142 men and 57 women		Other outcomes: perception	*******
			Mean age 57.36 (SD 6.8)		Questionnaire previously tested for reliability and validity	
Nagamma, T 2020^42^	To assess the health literacy about cervical cancer amongst the rural women in Udupi district, southern India, before and following intervention using audiovisual aid/face to face interactive sessions versus pamphlets alone	Pre-test-post-test	166 rural women visiting training centres	Cervical	Demographics, health literacy	100
India			Mean age 42.5 ± 2.5		Validated questionnaire	*******
Nguyen, M. H 2019^43^	To test the effects of a Web-based tailored educational intervention among newly diagnosed younger (<65 years) and older (≥65 years) patients with cancer	Randomised controlled study	232 patients categorized as younger (<65 years) and older (≥65 years) patients	General	Demographics, knowledge	86
Netherlands			158 men and 74 women		Other outcomes: website experience, information recall	******
			Mean age 63.50 (SD 9.06)		Netherlands patient information recall questionnaire	
Reder, M 2018^44^	To evaluate the effect of crowd-figure-pictograms on women’s numeric knowledge about mammography screening	Randomised controlled study	552 women aged 18 -49 (84.4%) 50-70 (15.6%)	Breast	Demographics, knowledge	71
UK			Mean age 37.5		Multiple choice questions	*****
Snyder, L 2022 5^45^	To assess the comprehension, utility, and preferences of longitudinal patient-reported outcome visualizations designed for prostate cancer survivors with limited literacy	Mixed methods	18 men	Prostate	Demographics, comprehension	100
USA			Mean age 69 (61-77)		Other outcomes: utility, preference	*******
					ISO 9186 standard for gist comprehension	
Trinh, N 2014^46^	To evaluate the effectiveness and patient satisfaction of a brief educational video on skin cancer risks and sun-protective behaviours in the transplant population during a routine posttransplant follow-up visit, as compared with an educational handout	Randomised controlled study	100 patients, 18 years and older	Skin	Demographics, knowledge	86
USA			Gender not specified		Other outcomes: patient satisfaction	******
			Age not reported		A 10-item questionnaire	
van Strien-Knippenberg, I. S 2024^47^	To investigate: 1) the effect of survival rates and side-effects presentation formats on comprehension and ‘feeling informed’; 2) differential effects among women with higher/lower health literacy, with adjuvant systemic breast cancer therapy as case example.	Randomised controlled study	501 Dutch women aged Mean age 59 years	Breast	Demographics, comprehension	86
Netherlands					Set of Brief Screening Questions (SBSQ) in Dutch	******
Volandes, A. E 2012^48^	To study whether a goals-of-care video would shape the choices made by patients with advanced cancer about their preferences for future medical care	Pre-post design	80 patients	Metastatic	Demographics, knowledge	86
USA			58 men and 22 women		Other outcomes: goals of care, preference	******
			Mean age 65 ± 12		6-question assessment	
Volk, R. J 2008^49^	To evaluate an entertainment-based patient decision aid for prostate cancer screening among patients with low or high health literacy.	Randomised controlled study	450 men aged 50 - 70 years	Prostate	Demographics, knowledge	71
USA			Mean age 55.6		Other outcomes: acceptability, engagement, decisional conflict, patient self-advocacy	*****
					Questions adapted from previous studies	
Wake, N 2019^50^	To investigate in the context of renal and prostate cancer the impact of using 3D printed and augmented reality models for patient education	Randomised controlled study	200 participants	Prostate and kidney	Demographics, understanding	43
USA			180 men and 2o women		5-point Likert scale survey	***
			Mean age, 63.64 ± 8.22			
Walker, M. S 2007^51^	To evaluate the educational intervention for objective and subjective knowledge, less anxiety, and better satisfaction with their care.	Randomised controlled study	122 Women	Breast	Demographics, knowledge	86
USA			Mean age 56	​	Other outcomes: patient satisfaction, state-trait anxiety inventory	******
					Questionnaire	
Wang, D. S 2015^52^	Video-based tool aimed at improving patient comprehension of key terminology related to prostate health.	Pre and post design	56 males	Prostate	Demographics, comprehension	86
USA			Mean age 56		Validated questionnaire	******
Zayed, A. M 2025^53^ USA	Impact of cervical cancer tool on health literacy	Pre and post design	95 women, 18 years and older	Cervical	Demographics, health literacy	86
			Mean age 32.4		Cervical cancer literacy assessment tool (C-CLAT) 18 knowledge questions	******

### Outcomes Measured

General outcomes measured in the studies varied across studies but mainly included cognitive, affective and behavioural domains, see Table 2.

Fifteen of the included studies assessed knowledge as an outcome, five measured comprehension, and one evaluated understanding. The areas of focus were screening, treatments, anatomical understanding, medical terminology and risk information in cancer. The studies employed different validated questionnaires to assess outcomes and only two studies primarily used a validated health literacy tool.^42,53^

Other outcomes considered included affective aspects including anxiety, worry, and decisional conflict, and behavioural domains such as screening or treatment intentions, role preferences, hypothetical decisions and choices for care levels. Self-efficacy and confidence in decision making and information use was another aspect frequently measured, see Table 2.

### Quality Assessment

The RCT studies met most of the MMAT quality criteria for RCTs. The most frequent reported limitation was lack of blinding in at least eight of the 11 RCTs^36,43,44,46,47,49-51^ and followed by lack of complete outcomes in three studies.^44,49,50^ Wake et al, (2019) scored the least in terms of MMAT quality due to lack of data on how randomization was performed, comparability of groups and it was also unclear whether blinding was performed.^50^ The lack of blinding for researchers or assistants may have introduced bias by influencing subjective outcomes reporting, and participants may have provided socially desirable responses. Additionally, unblinded research assistants involved in data collection may have unintentionally given more guidance or support to the intervention group, potentially affecting the validity of the results.^32,54^ The quantitative non-RCTs also met most quality criteria, particularly concerning clear objectives and appropriate measurement tools, but showed a significant lack of accounting for confounders. The three mixed methods studies met all the MMAT quality criteria for mixed method studies. Supplementary File 3 presents the results of the quality assessment of the included studies conducted using MMAT.

### Visual-Based Education Interventions Employed

Visual-based education interventions employed across studies included digital and audiovisual formats, such as video decision aids, interactive multimedia, tailored computer programs, web-based tailored information, and virtual reality educational interventions.^33-36,38,41,43,46,48,49,52,53^ Static print materials with visual components included informational brochures, pamphlets, illustrated flip charts, and paper-based decision aids with graphics and color-coded cards.^40,51^ Graphic designs and models included pictogram-based timelines, comics, photos, bar graphs, icon arrays, patient-reported outcome visualizations and 3D/augmented reality models.^37,39,44,45,47^

### Impact of Visual-Based Education Intervention on Knowledge

In the 15 studies reporting knowledge outcome, visual-based education interventions revealed positive effect in increasing knowledge across various health and screening topics, see Table 3. RCTs supported the impact of visual formats, finding videos to significantly increase skin cancer knowledge than written handouts,^46^ and the incorporation of crowd-figure-pictograms increased numeric mammography knowledge more effectively than numeric information alone.^44^ An RCT comparing different media types; videos, internet, and pamphlets found no difference of prostate cancer knowledge across interventions.^49^ Another RCT comparing three educational resources, a leaflet, a video, and an evidence-based booklet for men considering prostate cancer screening, revealed that while men receiving the video demonstrated a significant increase in knowledge scores post-intervention, this increase was less compared to evidence-based booklet which achieved significantly higher post-test knowledge scores.^36^

**Table 3.** Impact of Visual Interventions on Outcomes

Authors, year	Intervention and deliverer	Knowledge measures and tool	Findings
Cooper, E. C 2021,^33^	15 min educational video	Knowledge of human papilloma virus and cervical cancer	Increased knowledge 1.55/6 2.25 - 3.81/6
Pre post surveys	During screening session	6 MCQ knowledge Pre/Post video	**Note:** 26% no reading or writing, literacy level was approximately 73%, and average education level was equivalent to 7^th^ grade in the United States
Frosch, D. L 2008,^34^	Brief information brochure vs 30-minute video	Knowledge of prostate or colon cancer	The video decision aid group had significantly higher knowledge than the brochure group for both prostate (F (1,82) = 9.36, p = 0.003) and colon cancer (F (1,120) = 11.22, p = 0.001).
Quasi-experimental	Research assistant, physician and staff members	Questionnaire	For prostate cancer, knowledge increased by 21% in the English group and 13% in the Spanish group, while for colon cancer, the video group had 12% higher knowledge compared to the brochure group
			**Note:** Education levels showed that 35.3% in the brochure group and 25.5% in the decision aid group had an 8th-grade education or less
Gao, J 2022,^35^	30-minute virtual reality radiotherapy vs standard nursing care	Comprehension of radiotherapy	Change in comprehension (post-pre) for the intervention group was 28.10 (SD 10.80) compared to 1.57 (SD 5.90) for the control group; t (p value) =11.806 (<0.001)
RCT	Research team	Questionnaire with 14 statements	
Gattellari, M 2005,^36^	Leaflet vs 20-minute video vs booklet with visual aid	Composite knowledge of six domains of prostate cancer	The video group showed a greater pre-post knowledge increase than the Leaflet group (+5.0%) but a lesser gain than the Booklet group (-10.3%), with all differences statistically significant (p < 0.001).
RCT	Trained interviewers	14-item knowledge measure	**Note:** Information on knowledge was not modified by patient education level (F4,396 = 2.05, p = 0.09)
Housten, A. J 2020,^37^	Audio booklet with static pictographs vs video with static pictographs vs video with animated pictographs	Colorectal Cancer Screening Knowledge	No significant differences in total, verbatim, or gist knowledge across presentation formats, p > 0.05
RCT	Research assistants	Questionnaire with 14 questions in total	**Note:** Approximately one-quarter had inadequate health literacy (short test of functional health literacy in adult’s marginal/inadequate: 28%; brief health literacy screener low: 18%), and about half had low numeracy (subjective numeracy scale low: 54%; graphical literacy measures low: 50%).
Ilic, D 2008,^38^	20-minute video vs internet vs pamphlet	Knowledge of prostate cancer and screening	There were no statistically significant differences in mean knowledge between the video, internet, and pamphlet groups
RCT		A 5-item multiple-choice question	
Jambor, H. K 2025,^39^	Different information delivery formats (audio-only, audio with pictogram, audio with text)	Comprehension of cancer treatment paths	The proportion of correct responses was significantly greater for the audio-and-pictogram aid (16.5%) and for the audio-and-text aid (14.4%) compared with audio-only. The difference between pictogram and text aids was not significant
Mixed methods	Online	10 question questionnaires	
Lewis, C. L 2010,^40^	Paper version of decision aid vs educational component vs value clarification component	Colorectal cancer and screening Knowledge	Increased the proportion of participants with adequate knowledge from 4% to 52% (p < 0.01)
Pre-post design	Self-administered/research assistant	A 15-item true/false questionnaire	**Note:** Participants with adequate literacy were more likely to have adequate post-decision aid knowledge than those with inadequate or marginal literacy (64% vs 31% p = 0.04)
Menon, U 2008,^41^	A computer-based, theory-guided educational program called Tailored Messaging Intervention System vs no specific education	Knowledge of colorectal cancer and screening	Knowledge of colorectal cancer & screening intervention vs control; 0.64 (0.84) vs 0.91 (0.82)
Mixed methods	Research assistants	Questionnaire previously tested for reliability and validity	
Nagamma, T 2020,^42^	Audio-visual intervention followed by face-to-face interactive sessions vs pamphlet containing information on cervical cancer	Health literacy of cervical cancer	The intervention group (audio-visual) showed greater knowledge gains compared to the control across key cervical health literacy domains, personal hygiene (30.1%), risk factors (20.4%), symptoms (24.3%) and pap smear test knowledge (6.5%)
Pre-test-post-test	Health educator	Validated questionnaire	The increase in knowledge pre-and post-intervention in both groups
			was significant
			**Note:** The literacy rate was 100%, with education levels ranging from primary (38%) to graduate (18.6%)
Nguyen, M. H 2019,^43^	Intervention: mode-tailored website (enabling patients to tailor information using textual, visual, and audiovisual modes	Knowledge of cancer	In young participants, the mode-tailored website had slightly lower knowledge scores than text-only (-2.1) but higher scores than text+ images (+6.0) and text+ video (+1.7)
RCT	Control: non tailored website (text only, text with images, and text with videos)	Netherlands patient information recall questionnaire	In older participants, the mode-tailored website had similar knowledge scores to text-only (-0.5), slightly higher than text+ images (+1.3) and text+ video (+2.0)
	Researchers		
Reder, M 2018,^44^	Non-numeric information only vs numeric; non-numeric + numeric information vs crowd-figure pictogram; non-numeric + numeric information + crowd-figure	Knowledge of mammography screening	Women in the crowd-figure-pictogram group had a larger knowledge increase (2.42) than women in the numeric group (2.06), which was statistically significant (p = .03)
RCT	Online	Multiple choice questions	
Snyder, L 2022,^45^	4 prototypes (“Meter,” “Words,” “Comic,” and “Emoji”)	Gist and verbatim comprehension	Gist comprehension was highest with Meter (72.2%), followed by Emoji (55.6%), Comic (50.0%), and lowest with Words (33.3%), while verbatim comprehension was highest with Meter (83.3%), followed by Emoji (77.8%), Words (72.2%), and lowest with Comic (50.0%)
Mixed methods	Research team members	ISO 9186 standard for gist comprehension	**Note:** 78% had inadequate health literacy, and 89% had low graph literacy
Trinh, N 2014,^46^	2.01-minute education video vs 3-minute reading pamphlet	Knowledge acquisition	Video group showed a significantly greater improvement in knowledge scores (3.96 ± 1.69) compared to the pamphlet group (1.76 ± 1.42), with p < .001; diff =2.2
RCT	Researcher	A 10-item questionnaire	
van Strien-Knippenberg, I. S 2024,^47^	Experiment 1: different presentation formats (text block, bar graph, icon array) of survival rate	Gist and verbatim comprehension	Experiment 1:
RCT	Experiment 2 different presentation formats (survival format + no probability information, probability information in numbers without description, visualised probability information without description, probability information in numbers with accompanying description, visualised probability information with accompanying description) of side effect	Set of Brief Screening Questions (SBSQ) in Dutch	No significant effect of presentation format; Wald χ2 (2) = .83, p = .660, nor an interaction effect between format and health literacy on gist comprehension, Wald χ2 (2) = 2.74, p = .254., high health literacy associated with higher gist comprehension compared to those with low literacy, odds; 2.66 (95% CI, 1.28 to 5.55) times that of women with low health literacy
	Online		No significant effect of format on verbatim comprehension, Wald χ2 (2) = 2.15, p = .342, health literacy associated with verbatim comprehension, Wald χ2 (1) = 1.32, p = .251
			Experiment 2;
			No effect of format, Wald χ2 (4) = 4.68, p = .322, nor a significant interaction between format, health literacy influence gist comprehension, Wald χ2 (1) = .61, p = .436
Volandes, A. E 2012,^48^	6-minute video	Knowledge about goals of care choices	Participants significantly improved their knowledge scores after viewing the video compared to baseline (mean change: 1.6 ± 0.95; P < .001)
Pre-post design	Research assistant	Measured with a 6-question assessment	
Volk, R. J 2008,^49^	Entertainment-based decision aid: a computerized, interactive, multimedia aid with an entertaining soap-operatic component vs audio booklet-control aid: contained the same factual content as the entertainment-based aid, but lacked interactivity	Knowledge of prostate cancer and screening	Subjects from the low-literacy site had lower knowledge scores than subjects from the high literacy site. However, subjects from both sites had significant improvements in knowledge regardless of the decision aid they received and there were no significant differences between the aids in subjects’ knowledge gains
RCT	Research assistants	Questions adapted from previous studies	**Note:** Patients at the low-literacy site were more engaged with the entertainment-based aid than patients at the high-literacy site
Wake, N 2019,^50^	3D printed and augmented reality kidney and prostate cancer models	Comprehension and understanding of cancer	Patients had a greater understanding using 3D printed models versus imaging for all measures including comprehension of disease, cancer size, cancer location, treatment plan, and the comfort level regarding the treatment plan (range 4.60-4.78/5 vs. 4.06-4.49/5, p < 0.05)
RCT	Surgeon	5-point Likert scale survey	
Walker, M. S 2007,^51^	Educational intervention consisted of a 47-page flip chart	Knowledge of breast biopsy	No statistical difference between control and intervention. Useful for English second language
RCT	Research assistant	Questionnaire	
Wang, D. S 2015,^52^ USA	Animated video based educational tool	Comprehension of prostate health terminology	Video based decisional aides improved comprehension of prostate health term by 68%
Qualitative	Researchers	Validated questionnaire	
Zayed, A. M 2025,^53^ USA	Online cervical cancer patient education tool	Cervical cancer literacy assessment tool (C-CLAT) 18 knowledge questions	Tool increased awareness in non-English-speaking migrants in USA
Pre and post design	Online		

Conversely, the non-RCTs consistently reported clear knowledge benefits: video decision aids improved cancer screening knowledge for ethnically diverse patients compared to brochures^34^ and significantly increased advanced cancer goals-of-care knowledge post-intervention.^48^ Furthermore, quasi-experimental designs indicated that videos significantly improved cervical cancer knowledge in mass screening participants in Tanzania.^33^ Audio-visual sessions increased knowledge among rural Indian women^42^ and increased cervical cancer literacy in Arabic-speaking women.^53^ Also, a targeted decision aid utilizing graphics increased knowledge about colorectal cancer screening in older adults.^40^

Mixed methods studies with a randomised controlled intervention component, including one comparing video, internet, and written materials^38^ and another comparing animated vs. static pictographs,^37^ found no statistically significant difference in knowledge scores between visual and non-visual presentations. Nguyen et a al 2024 a RCTs, found that intervention mode tailoring did not significantly increase knowledge before consultation, although higher pre-consultation knowledge predicted better information recall.^43^ In another study, although an educational flip chart intervention found no significant difference between groups, it enhanced objective and subjective knowledge for African American patients undergoing breast biopsy.^51^

### Meta-Analysis Results

A meta-analysis was conducted for the RCT studies that provided relevant quantitative data on knowledge outcomes. Gao J (2022)^35^ study labelled the outcome as comprehension, the authors stated that the tool used in the comprehension section specifically measured patient’s knowledge of radiotherapy, providing the quantitative data required for inclusion in the meta-analysis. For studies that did not report within-group mean ± SD differences, the mean change was calculated as the difference between post- and pre-intervention scores. The standard deviation of the mean change was then derived using the formula:

SD = √(SD^2^pre + SD^2^post - 2r × SDpre × SDpost), where the correlation coefficient (r) between pre- and post-intervention scores was assumed to be 0.5.

**Figure 2.**
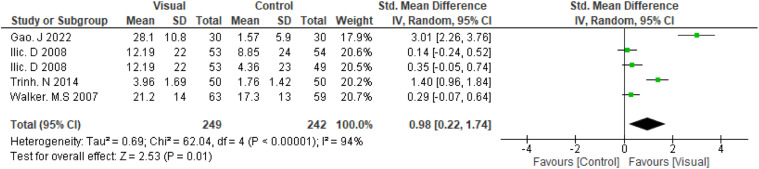
Meta-analysis for RCTs

**Figure 3.**
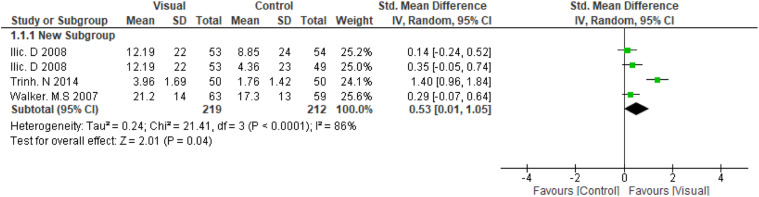
Subgroup analysis of conventional visual-based education interventions

**Figure 4.**
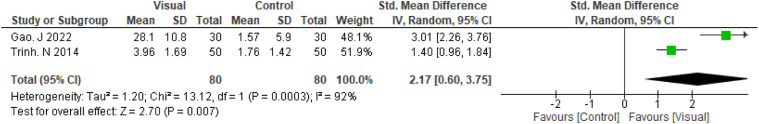
Sub-group analysis of the two studies that reported within group mean difference and SD

The overall pooled effect estimate was 0.98 (95 % CI: 0.22, 1.74), see Figure 2, indicating a statistically significant increase in knowledge in favour of visual-based education. However, there was substantial heterogeneity across the included studies (I^2^ = 94%). A subgroup analysis was performed by excluding the study by,^35^ in which the intervention involved 3D virtual reality simulation, a more interactive and potentially more impactful intervention compared with conventional visual-based educational intervention used in the other studies. The resulting pooled estimate was 0.53 (95% CI: 0.01, 1.05), see Figure 3, showing that visual interventions continued to have a positive effect on knowledge, although the confidence interval crossed the line of no effect and heterogeneity remained high (I^2^ = 86%). A further subgroup analysis, limited to the two studies that reported within-group mean differences, yielded a pooled estimate of 2.17 (95% CI: 0.60, 3.75), I^2^ = 92%, see Figure 4. Subgroup analyses by cancer type, educational tool, or intervention duration could not be performed due to the small number and diversity of the included studies. The four included studies varied widely in cancer type, tool used, and exposure time, making it difficult to identify specific sources of heterogeneity or conduct reliable statistical comparisons.

### Impact of Visual-Based Education Intervention on Comprehension and Understanding

Studies that assessed the impact of interventions on comprehension show mixed results across study designs: RCTs demonstrated that visual-based treatment timelines significantly improved participant correct responses compared to audio-only information,^39^ and a Virtual Reality system significantly increased radiotherapy comprehension scores compared to standard care.^35^ Another RCT comparing visual formats (bar graphs, icon arrays) to a well-designed numerical text block found no enhancement in gist or verbatim comprehension regarding breast cancer survival rates.^47^ Non-RCT studies consistently reported positive impacts, as a video-based tool significantly improved comprehension of prostate health terms by 68%.^52^ In another non-RCT that studied longitudinal patient-reported outcome prototypes, Meter (visual analogy of size or time) achieved the best gist comprehension and Emoji had the highest verbatim comprehension, whereas Comic had the poorest comprehension.^45^

One study evaluated the impact of intervention on understanding and findings showed that 3D printed models increased patient understanding of their anatomy, disease, tumour characteristics, and surgical procedure compared to imaging alone.^50^

### Association Between Baseline Health Literacy and Outcomes

Nine studies assessed health literacy as a baseline characteristic that moderates how visual-based education interventions impact knowledge and comprehension,^33,37,39,40,43,45,47,49,52^ see Table 4. In two studies related to cervical cancer education, health literacy was measured pre- and post-intervention, making it a primary outcome to measure the effectiveness of the tool itself.^42,53^ Health literacy was broadly defined, often encompassing the ability to obtain, process, and understand basic health information and services for appropriate decisions^37,43,47,53^ or, more specifically, skills beyond basic reading and writing, such as numeracy, speech comprehension, and conceptual knowledge.^45,49^ Tools used for measurement include the Short Test of Functional Health Literacy in Adults,^37,40^ the Newest Vital Sign,^45^ the Rapid Estimate of Adult Literacy in Medicine^52^ and condition-specific measures like the cervical cancer literacy assessment tool^53^ or the Short Assessment of Health Literacy in Dutch.^43^

**Table 4.** Association Between Baseline Health Literacy and Outcomes

Authors, year	Health literacy definition	How health literacy was measured	Tool used	Association
Cooper, E. C 2021,^33^	Not defined	Literacy was generally reported as a binary feature (e.g., ability to read/write). Reading ability was reported as Yes (73.7%) or No (26.2%)	Demographic survey	Post-video scores significantly improved for all participants regardless of literacy. However, multivariate analysis identified reading ability as a statistically significant factor in the change of score (p=.0028)
Tanzania.				Participants classified as having adequate health literacy (S-TOFHLA scores) had significantly different gist knowledge scores depending on the intervention arm they received (p= 0.01)
Housten, A. J 2020,^37^	The degree to which individuals have the capacity to obtain, process, and understand basic health information and services needed to make appropriate health decisions	Participants were classified into 3 levels based on their scores: ‘‘inadequate’’ (0–16), ‘‘marginal’’ (17–22), and ‘‘adequate’’ (23– 36) health literacy	Short Test of Functional Health Literacy in Adults (S-TOFHLA), Brief Health Literacy Screener (BHLS), Subjective Numeracy Scale (SNS) (for numeracy), and Graphical Literacy Measure (GLM) (for graphical literacy)	The intervention format did not have a statistically significant effect on total, verbatim, or gist knowledge scores, even when categorised by health literacy and numeracy levels
USA				
Jambor, H. K 2025,^39^	The ability to understand written and verbal medical information about diagnosis, prognosis, uncertainties, and risks, which is important for shared decision-making	Participants in Study 1 were categorized as having high health literacy (95%) or low health literacy (5%)	The participants’ health literacy was tested with 3 control questions to monitor a possible selection bias (validated test from a previous study	The cohort was relatively homogeneous, with over 95% demonstrating high health literacy. Consequently, the researchers could not reliably test differences in visual representation transparency and translucency across literacy levels
Germany				
Lewis, C. L 2010,^40^	Not defined	Categorized as adequate, marginal, and inadequate	Short Test of Functional Health Literacy in Adults (S-TOFHLA) was administered	Participants with adequate literacy were more likely to have adequate post-decision aid knowledge than those with inadequate or marginal literacy (64% vs 31% p = 0.04) and were more likely to be prepared to make an individualized decision after decision aid use: (54% of those with adequate literacy met the criteria, vs. 19% of those with inadequate or marginal literacy p = 0.03)
USA				
Nagamma, T 2020,^42^	Not defined	Knowledge was measured based on the scores (frequencies and percentages) from the questionnaire	A validated questionnaire was used to assess knowledge pre- and post-intervention	The literacy rate in women was 100%, out of which 38% had primary education, 43.4% had secondary level of education and 18.6% of the women completed their graduation
India				Knowledge related to cervical cancer was quite low prior to the intervention. Following intervention knowledge improved significantly in both the experimental and control groups across several variables (p < 0.001)
Nguyen, M. H 2019,^43^ Netherlands	The capacity to obtain, process, and understand basic health information needed to make appropriate decisions	Health literacy was measured as a continuous sum score of correct answers on 22 health-related words, potentially ranging from 0 to 22	Comprehension test of the Short Assessment of Health Literacy in Dutch (SAHL-D)	Health literacy was found to significantly relate to website knowledge when included in analyses
Snyder, L 2022,^45^	An understanding of words (prose), numbers (numeracy), and forms (documents)	Health literacy measured by NVS results classified participants into three categories: Adequate literacy (22.2%), possibility of limited literacy (33.3%), or High likelihood of limited literacy (44.4%	Newest Vital Sign (NVS), a validated 6-item assessment tool, was used to measure health literacy. The Short Graph Literacy scale (SGL) was used to assess graph literacy	The study focused on whether certain visualization prototypes (Meter, Words, Comic, Emoji) performed well among this primarily limited literacy population. The Meter and Emoji prototypes performed well for comprehension, utility, and preference. The Comic prototype performed notably worse, potentially because its text component required more reading, suggesting literacy challenges influenced its comprehension
USA		The majority of participants had less than a college degree (95%), had inadequate health literacy (78%), and low graph literacy (89%)		
van Strien-Knippenberg, I. S 2024,^47^	Accessing, understanding, and using health information	Health literacy was assessed before the experiments with the Set of Brief Screening Questions (SBSQ), a self-reported measure with three questions measured on a 5-point scale (0 = always, 4 = never). Women who scored ≤2 on one of the questions were indicated as having lower health literacy.	Set of Brief Screening Questions (SBSQ) in Dutch was used	Higher health literacy was associated with higher gist comprehension of survival rates. The odds of women with high health literacy having higher gist comprehension were 2.66 times greater than women with low health literacy. None of the presentation formats could overcome these health literacy differences in comprehension
Netherlands				
Volk, R. J 2008,^49^	According to the Institute of Medicine, health literacy includes skills beyond reading and writing – such as numeracy, speech and speech comprehension, and basic math calculation - and relies on cultural and conceptual knowledge	No direct assessment: health literacy inferred based on clinical setting	No tool used	Patients at the low-literacy site were more engaged with the entertainment-based aid than patients at the high-literacy site. Overall, knowledge improved for all patients. Among patients at the low literacy site, the entertainment-based aid was associated with lower decisional conflict and greater self-advocacy
USA		Low health literacy site was a general medicine clinic in a publicly funded hospital operated by a county health district		Overall, subjects from the low-literacy site had lower knowledge scores than did subjects from the high-literacy site. Nevertheless, subjects from both sites had significant improvements in
		High health literacy site) was a university-affiliated family medicine clinic that serves a generally well-educated and privately insured patient population		knowledge
Wang, D. S 2015,^52^	Not defined	At test was used to compare improvements in comprehension between low-literacy patients (REALM score < 60) and high-literacy patients (REALM score 60).	Rapid Estimate of Adult Literacy in Medicine (REALM)	Low literacy patients demonstrated statistically significant improvements in the comprehension p = .106
USA				
Zayed, A. M 2025,^53^ USA	The degree to which individuals can find, understand, and use information and services to inform health-related decisions and actions for themselves and others	The 22-item tool assesses the 3 cervical cancer content domains: (1) cervical cancer awareness, (2) cervical cancer knowledge and screening, and (3) prevention and control. Each content domain’s score was calculated using the mean of pertaining questions	Cervical cancer literacy assessment tool (C-CLAT) 18 knowledge questions	Health literacy increased overall after self-administration of the tool, specifically in terms of cervical cancer prevention and control (p<0.01)

Health literacy was categorised as low/inadequate or high/adequate literacy levels.^37,40,47,52^ The association between health literacy and outcomes showed varied results regarding intervention effectiveness: an entertainment-based decision aid, inferred to be targeted at a low literacy site led to lower decisional conflict and greater self-advocacy for prostate cancer screening compared to a control audio booklet in that group, whereas no such difference was found at the high literacy site.^49^ In Volk et al.^49^ literacy was inferred from clinic location rather than measured for each patient, as some patients at the low-literacy clinic may actually have had high literacy, and vice versa, which may have affected the results. Differences in patient characteristics, such as education, income, and insurance, could also have influenced the findings A video-based educational tool improved patient comprehension of prostate health terminology effectively regardless of literacy level^52^ and video education enhanced cervical cancer knowledge independent of literacy, although reading ability was a statistically significant factor in the magnitude of score change in multivariate analysis.^33^ For visualization of patient-reported outcomes among a population with predominantly limited literacy, optimized graphics like the Meter and Emoji prototypes had better outcomes for comprehension.^45^ However, comparisons involving formats for communicating complex data often found no significant differences in comprehension improvement across visual and well-designed numerical textual formats for survival, despite the lack of format advantage, baseline health literacy remained critical, as higher health literacy was associated with higher gist comprehension of survival information^47^ and those with adequate literacy were more likely to be prepared for individualized decision-making than those with inadequate literacy regarding colorectal cancer screening.^40^ Conversely, interventions designed to increase knowledge successfully resulted in a statistically significant increase in overall health literacy scores, especially in awareness, prevention and control domains.^53^

## Discussion

This systematic review studied the effectiveness of visual-based education interventions on understanding cancer education focusing on knowledge, comprehension and understanding of the information. Visual-based interventions presented in various formats were used across different cancer types, including prostate cancer,^34,36,38,45,49,52^ colorectal cancer screening,^37,40,41^ breast cancer screening, biopsy consent and adjuvant therapy decisions,^44,47,51^ and cervical cancer screening and human papillomavirus education^33,42,53^ alongside interventions for advanced cancer goals of care.^48^ The target populations were often characterized by vulnerability, including low-literacy patients and ethnically diverse groups (African American, Latino, Arabic-speaking immigrants), as well as the elderly making individualized screening decisions.^34,40,49,51-53^ Intervention components ranged from basic formats like illustrated flip charts and pictogram enhanced print materials to complex multi-modal digital tools such as video decision aids, interactive mode-tailored websites, and virtual models like 3D printed anatomy models and virtual reality simulators.^34,44,51^

The findings revealed that structured educational videos and multimedia platforms enhanced understanding of complex medical concepts and terminology of cancer prevention and treatment compared to traditional brochures, thereby supporting informed decision-making.^34,48,49^ For instance, a video-based educational intervention significantly improved baseline knowledge of human papilloma virus and cervical cancer among women in Tanzania, independent of their existing education or literacy level.^33^ Similarly, patients with advanced cancer demonstrated significantly improved knowledge scores after viewing an educational video on goals of care and in a study comparing video versus pamphlets for skin cancer education, the video group showed a significantly greater improvement in knowledge scores.^46,48^ In studies that employed specific visual aids, such as pictogram-based treatment timelines or crowd-figure-pictograms to illustrate population risk, effectively conveyed numeric leading to improved gist and verbatim comprehens^51^ ion and recall.^39,44,45^ The focus on visualization addresses the limitations of low numeracy and reading literacy, leading to higher reported confidence and satisfaction especially in awareness, prevention and control domains.^45,49,53^ Although visual formats generally enhanced engagement and clarity, the effectiveness of visual aids over well-designed, structured text remains context-dependent, with some studies finding equally effective or even higher knowledge gains using comprehensive written materials for certain screening information.^36,38,47^ Text-based handouts can seem to perform better than video-based when they use clear structure, evidence-based content, plain language, and decision-support exercises.^39,44,46,47^ Conversely, visual tools sometimes fail to provide added benefits due to shallow information processing, content redundancy, or being perceived as inappropriate for serious medical topics.^36,43,49^ Additionally, the topic may have been relatively simple for the participants with high baseline knowledge creating a ceiling effect that limited further improvement.^51,52^ Ultimately, integrating visuals improves information readability, empowering patients in shared decision-making processes by making complex information more easily processed and retained.^39,48^

Previous systematic reviews have shown consistent benefits of visual-based education interventions on knowledge and comprehension,^3,15,16,55^ particularly focused for people with lower baseline health literacy or numeracy.^15,56^ Visualised information shows promising improvements on understanding of complex risk information and treatment goals for decision making.^56^ Interventions designed to enhance understanding across varying health literacy levels can improve illness comprehension and promote more appropriate behaviours, including participation in cancer screenings, adherence to complex therapies, engagement in treatment, and effective self-management of everyday health.^3^ Visual-based education interventions are beginning to become a focused strategy to communicate complex cancer information to patients, caregivers and the public. Visualised education represents a compelling method for improving patient knowledge and comprehension, critical components of health literacy, especially among populations facing educational or linguistic barriers.^3,15,33,52,55^

As noted in previous studies,^3,10,57^ health literacy was defined variably across studies, typically emphasizing the ability to access, understand, and use health information.^47,53^ For instance, one conceptualization views health literacy broadly, including reading, writing, numeracy, speech comprehension, and conceptual knowledge,^49^ while another defines personal health literacy as the ability to find, understand, and use information to inform health-related decisions.^53^ The focus in several studies is on the individual’s ability to understand and interpret abstract health information.^47^ Health literacy was measured using diverse tools: The Short Test of Functional Health Literacy in Adults and the Brief Health Literacy Screener were used to categorize literacy levels,^37,43^ while the Rapid Estimate of Adult Literacy in Medicine measured patient reading levels.^52^ Furthermore, self-reported measures like the Set of Brief Screening Questions were employed to assess the frequency of difficulty understanding written medical information.^47^

Health literacy was a significant moderator of intervention outcomes, low health literacy was consistently associated with worse baseline knowledge and gist comprehension of concepts like survival rates, regardless of visualization format.^45,47,52^ Multimedia interventions tailored to low-literacy audiences can mitigate these disparities, leading to improvements in comprehension and increased self-advocacy.^49,58^ In one case, women with lower health literacy felt better informed than high-health literacy women when provided with visualized probability information without accompanying descriptions, demonstrating complex interaction effects on subjective measures.^47^

## Limitations

The review is not without limitations, firstly although a meta-analysis was conducted, substantial heterogeneity persisted across the included studies and could not be fully explained, despite efforts to explore potential sources through subgroup analyses. As a result, the findings should be interpreted with caution and underscore the need for further investigation. While the MMAT quality assessment indicated that the included studies were generally of good quality, most RCTs lacked assessor blinding, potentially introducing bias and affecting the validity of outcome assessments. Furthermore, there was an inadequate adjustment for confounding variables, which is essential to identify factors that may influence the observed effects of the intervention in non-RCTs. Additionally, the methodology for health literacy measurement is inconsistent, limiting direct cross-study comparisons of health literacy findings. However, studies demonstrated that knowledge, comprehension, and understanding are essential components of health literacy as measured by outcomes directly related to processing and interpreting health information. Although health literacy was rarely the primary outcome, its measurement using diverse tools like Short Test of Functional Health Literacy in Adults, Rapid Estimate of Adult Literacy in Medicine and Single Item Literacy Screener allowed researchers to assess its crucial role as a moderator, revealing that low health literacy is consistently associated with lower comprehension and knowledge. Consequently, success in improving patient knowledge and comprehension through visual education is seen as a direct way to mitigate the barriers imposed by limited health literacy, enabling more informed decision-making across various cancer contexts.

## Conclusion

Overall, the findings of this review support the concept that visual-based education enhances knowledge, understanding, and comprehension of cancer prevention and treatment, particularly in relation to health literacy. This, in turn, empowers individuals to access and interpret health information more effectively, facilitates informed decision-making, and improves their ability to navigate the healthcare system.

## Supplemental Material

Supplemental Material - Effectiveness of Visual-Based Interventions on Understanding Cancer Information: A Systematic Review Supplemental Material for Effectiveness of Visual-Based Interventions on Understanding Cancer Information: A Systematic Review by Linda Siachalinga, Hansoo Kim, Karin Brochstedt Dieperink, and Elisabeth Coyne in Cancer Control.

Supplemental Material - Effectiveness of Visual-Based Interventions on Understanding Cancer Information: A Systematic Review Supplemental Material for Effectiveness of Visual-Based Interventions on Understanding Cancer Information: A Systematic Review by Linda Siachalinga, Hansoo Kim, Karin Brochstedt Dieperink, and Elisabeth Coyne in Cancer Control.

Supplemental Material - Effectiveness of Visual-Based Interventions on Understanding Cancer Information: A Systematic Review Supplemental Material for Effectiveness of Visual-Based Interventions on Understanding Cancer Information: A Systematic Review by Linda Siachalinga, Hansoo Kim, Karin Brochstedt Dieperink, and Elisabeth Coyne in Cancer Control.

## Appendix

### Abbreviations

PRISMA: Preferred Reporting Items for Systematic Reviews and Meta-Analyse
RCTs: Randomised Controlled Trials
MMAT: Mixed Methods Appraisal Tool
