# Supplementary material for: Effectiveness of Visual-Based Interventions on Understanding Cancer Information: A Systematic Review
Source: Cancer Control. 2026 Apr 27;33:10732748261446035. doi: 10.1177/10732748261446035 (PMC13133456; doi:10.1177/10732748261446035)
Supplement: Supplemental Material - Effectiveness of Visual-Based Interventions on Understanding Cancer Information: A Systematic Review [file sj-pdf-2-ccx-10.1177_10732748261446035.pdf]

**Search strategy**

**PubMed**

| Search number | Query |
|---|---|
| 4 | ((("visual*"[All Fields] OR "visual intervention*"[All Fields] OR "visual aid*"[All Fields] OR "infographic*"[All Fields] OR "pictogram*"[All Fields] OR "visual communicat*"[All Fields] OR "visual education"[All Fields] OR "visual message*"[All Fields])) AND ("health literacy" OR "patient understanding" OR "comprehension" OR "patient education")) AND ("cancer" OR "oncology" OR "neoplasm" OR "tumor" OR "carcinoma") |
| 3 | "cancer" OR "oncology" OR "neoplasm" OR "tumor" OR "carcinoma" |
| 2 | "health literacy" OR "patient understanding" OR "comprehension" OR "patient education" |
| 1 | ("visual*"[All Fields] OR "visual intervention*"[All Fields] OR "visual aid*"[All Fields] OR "infographic*"[All Fields] OR "pictogram*"[All Fields] OR "visual communicat*"[All Fields] OR "visual education"[All Fields] OR "visual message*"[All Fields]) |

**Embase**

| | |
|---|---|
| #4 | #1 AND #2 AND #3 |
| #3 | 'cancer'/exp OR 'oncology'/exp OR 'neoplasm'/exp OR 'tumor':ab,ti OR 'carcinoma'/exp |
| #2 | 'health literacy'/exp OR 'patient understanding':ab,ti OR 'comprehension':ab,ti OR 'patient education'/exp |
| #1 | 'visual*':ab,ti OR 'visual intervention*':ab,ti OR 'visual aid*':ab,ti OR 'infographic*':ab,ti OR 'pictogram*':ab,ti OR 'visual communication*':ab,ti OR 'visual education':ab,ti OR 'visual message*':ab,ti |

**Scopus**

| |
|---|
| ( TITLE-ABS-KEY ( visual OR visual intervention OR visual aid OR infographic OR pictogram OR visual communication OR visual education OR visual message ) AND TITLE-ABS-KEY ( health literacy OR patient understanding OR comprehension OR patient education ) AND TITLE-ABS-KEY ( cancer OR oncology OR neoplasm OR tumor OR carcinoma ) ) |
