# Supplementary material for: Effectiveness of Visual-Based Interventions on Understanding Cancer Information: A Systematic Review
Source: Cancer Control. 2026 Apr 27;33:10732748261446035. doi: 10.1177/10732748261446035 (PMC13133456; doi:10.1177/10732748261446035)
Supplement: Supplemental Material - Effectiveness of Visual-Based Interventions on Understanding Cancer Information: A Systematic Review [file sj-pdf-3-ccx-10.1177_10732748261446035.pdf]

Supplementary data

Key	
Yes	🟢
No	🔴
Can't tell	🟡

Randomized Controlled Studies

AUTHOR/YEAR	TITLE	MMAT Score	S1. Are there clear qualitative and quantitative research questions (or objectives*)	S2. Do the collected data allow address the research question (objective)?	2.1. Is randomization appropriately performed?	2.2. Are the groups comparable at baseline?	2.3. Are there complete outcome data?	2.4. Are outcome assessors blinded to the intervention provided?	2.5. Did the participants adhere to the assigned intervention?
Gao, J 2022	Pilot Study of a Virtual Reality Educational Intervention for Radiotherapy Patients Prior to Initiating Treatment	100	🟢	🟢	🟢	🟢	🟢	🟢	🟢
Gattellari, M 2005	A community-based randomised controlled trial of three different educational resources for men about prostate cancer screening	86	🟢	🟢	🟢	🟢	🟢	🔴	🟢
Housten, A. J 2020	Does Animation Improve Comprehension of Risk Information in Patients with Low Health Literacy? A Randomized Trial	100	🟢	🟢	🟢	🟢	🟢	🟢	🟢
Ilic, D 2008	Informing Men about Prostate Cancer Screening: A Randomized Controlled Trial of Patient Education Materials	100	🟢	🟢	🟢	🟢	🟢	🟢	🟢
Nguyen, M. H 2019	Tailored web-based information for younger and older patients with cancer: randomized controlled trial of a preparatory educational intervention on patient outcomes	86	🟢	🟢	🟢	🟢	🟢	🟡	🟢
Reder, M., & Thygesen, L. C 2018	Crowd-figure-pictograms improve women's knowledge about mammography screening: results from a randomised controlled trial.	71	🟢	🟢	🟢	🟢	🟡	🟡	🟢
Trinh, N 2014	Use of a brief educational video administered by a portable video device to improve skin cancer knowledge in the outpatient transplant population	86	🟢	🟢	🟢	🟢	🟢	🔴	🟢
van Strien-Knippenberg, I. S 2024	Presenting decision-relevant numerical information to Dutch women aged 50–70 with varying levels of health literacy: Case example of adjuvant systemic therapy for breast cancer	86	🟢	🟢	🟢	🟢	🟢	🟡	🟢
Volk, R. J 2008	Entertainment education for prostate cancer screening: a randomized trial among primary care patients with low health literacy	71	🟢	🟢	🟢	🟢	🟡	🔴	🟢
Wake, N 2019	Patient-specific 3D printed and augmented reality kidney and prostate cancer models: impact on patient education	43	🟢	🟢	🟡	🟡	🟡	🟡	🟢
Walker, M. S 2007	Educational intervention for women undergoing image-guided breast biopsy: results of a randomized clinical trial. Cancer Control,	86	🟢	🟢	🟢	🟢	🟢	🔴	🟢
	**Average score**	**83**							

Non-Randondomized Controlled Studies

AUTHOR/YEAR	TITLE	MMAT Score	S1. Are there clear qualitative and quantitative research questions (or objectives*)	S2. Do the collected data allow address the research question (objective)?	3.1. Are the participants representative of the target population?	3.2. Are measurements appropriate regarding both the outcome and intervention (or exposure)?	3.3. Are there complete outcome data?	3.4. Are the confounders accounted for in the design and analysis?	3.5. During the study period, is the intervention administered (or exposure occurred) as intended?
Cooper, E. C 2021	Implementation of human papillomavirus video education for women participating in mass cervical cancer screening in Tanzania.	100	🟢	🟢	🟢	🟢	🟢	🟢	🟢
Frosch, D. L 2008	Using decision aids in community-based primary care: A theory-driven evaluation with ethnically diverse patients	100	🟢	🟢	🟢	🟢	🟢	🟢	🟢
Lewis, C. L 2010	A targeted decision aid for the elderly to decide whether to undergo colorectal cancer screening: development and results of an uncontrolled trial	86	🟢	🟢	🟢	🟢	🟢	🟡	🟢
Nagamma, T 2020	Effectiveness of audio-visual and print media intervention on knowledge of cervical health among rural women in southern india	100	🟢	🟢	🟢	🟢	🟢	🟢	🟢
Volandes, A. E 2012	Augmenting advance care planning in poor prognosis cancer with a video decision aid	86	🟢	🟢	🟢	🟢	🟢	🔴	🟢
Wang, D. S 2015	Video-based educational tool improves patient comprehension of common prostate health terminology.	86	🟢	🟢	🟢	🟢	🟢	🟡	🟢
Zayed, A. M 2025	Improving cervical cancer health literacy in Arabic-speaking immigrant women in the United States through an online patient education tool	86	🟢	🟢	🟢	🟢	🟢	🟡	🟢
	**Average score**	**92**							

Mixed methods

AUTHOR/YEAR	TITLE	MMAT score	S1. Are there clear qualitative and quantitative research questions (or objectives*)	S2. Do the collected data allow address the research question (objective)?	5.1. Is there an adequate rationale for using a mixed methods design to address the research question?	5.2. Are the different components of the study effectively integrated to answer the research question?	5.3. Are the outputs of the integration of qualitative and quantitative components adequately interpreted?	5.4. Are divergences and inconsistencies between quantitative and qualitative results adequately addressed?	5.5. Do the different components of the study adhere to the quality criteria of each tradition of the methods involved?
Jambor, H. K 2025	Communicating cancer treatment with pictogram-based timeline visualizations	100	●	●	●	●	●	●	●
Menon, U 2008	Interactive, culturally sensitive education on colorectal cancer screening	100	●	●	●	●	●	●	●
Snyder, L 2022	Comprehension, utility, and preferences of prostate cancer survivors for visual timelines of patient-reported outcomes co-designed for limited graph literacy: meters and emojis over comics	100	●	●	●	●	●	●	●
	**Average score**	**100**							
